# Editorial: Precision Physical Activity and Exercise Prescriptions for Disease Prevention: The Effect of Interindividual Variability Under Different Training Approaches, Volume II

**DOI:** 10.3389/fphys.2021.831403

**Published:** 2022-01-14

**Authors:** Robinson Ramírez-Vélez, Mikel Izquierdo

**Affiliations:** ^1^Navarrabiomed, Hospital Universitario de Navarra (HUN), Navarra Institute for Health Research (IdiSNA), Universidad Pública de Navarra (UPNA), Pamplona, Spain; ^2^Centro de Investigación Biomédica en Red de Fragilidad y Envejecimiento Saludable (CIBERFES), Instituto de Salud Carlos III, Madrid, Spain

**Keywords:** interindividual variability, precision physical activity, dose-response, disease prevention, health-related quality of life

The second volume of the Research Topic entitled “*Precision Physical Activity and Exercise Prescriptions for Disease Prevention: The Effect of Interindividual Variability Under Different Training Approaches*” has been successfully completed, as expected. As stated in the preface to the first volume, this Research Topic was initially intended to address a challenge in this field, but this topic is becoming, over time, an important cornerstone for scientists who are exploring the fascinating subject of “Precision Physical Activity and Exercise Prescriptions for Disease Prevention” (Ramírez-Vélez et al., 2017). This Research Topic consists of 10 articles, of which seven contain original data, one is a systematic review with meta-analysis and two are opinion/hypothesis articles.

We open the second volume of this with an interesting hypothesis and theory article described by Gentil et al., from the Federal University of Goiás, Brazil. The manuscript presents a useful overview of practical recommendations relevant to the use of resistance training for people who have been diagnosed with COVID-19 during different phases of disease, with a special focus on immune, respiratory, and cardiovascular systems.

Lazarus and Harridge's opinion article proposes a novel hypothesis called “The Interplay of Exercise and Physiological Heterogeneity as Drivers of Human Aging.” Both authors discuss this hypothesis and related concepts in the context of the trajectory of healthy and non-healthy human aging. Here, the human aging process, interacting with lifestyle factors and heterogeneous physiologies, governs and modifies all processes in all humans all the time, yet its presence does not seem to merit a place in most physiology or medical texts.

de Santana et al., call for action of alternative assessments to understand the variability of the response and assist in planning new studies. The authors suggested that such a range could be based on the number of sets per muscle group, comprising at least 10 sets per week. Additionally, Omic sciences (e.g., transcriptomics, epigenomics, proteomics, and/or metabolomics) emerge as the next frontier to be explored in this field of knowledge that help to explain the complexity to phenomenon of exercise response heterogeneity.

Bonafiglia et al., systematic review lays the groundwork of the Research Topic, to determine the extent to which studies in the exercise training literature have adopted sound statistical approaches for examining individual responses to exercise training. Bonafiglia underlines the compelling need for prospective trials to identify studies that statistically estimated the presence of interindividual differences in trainability substantially in participant characteristics, training modes, and outcomes assessed. The authors addressed novel data better convince researchers to statistically estimate interindividual differences in trainability and consider error and an smallest worthwhile change or minimum clinically important difference in future clinical trials.

Castro et al., report that metabolic profile and pathways in blood serum and the skeletal muscle responses after 8-week of continuous endurance training (ET) or high-intensity interval training (HIIT), in a group of 70 men, young and sedentary. The main finding changed and impacted pathways by these metabolites were: arginine and proline metabolism, glycine, serine and threonine metabolism, and glyoxylate and dicarboxylate metabolism for both ET and HIIT programs; and additional alanine, aspartate and glutamate metabolism, arginine biosynthesis, glycolysis/gluconeogenesis, and pyruvate metabolism for ET. These results suggest that regulating the metabolism of amino acids and carbohydrates may be a potential mechanism for understanding the inter-individual variability of cardiorespiratory fitness in responses to ET and HIIT programs.

Gallegos-Carrillo et al., showed for the first time a pragmatic cluster randomized trial, in 4 Primary Health Care Units. Differences were observed in triglycerides, BMI, metabolic risk scores variables and depressive symptoms among exercise referral and brief physical activity counseling programs. In addition, differences in the brief physical activity group were observed according to level of adherence in blood pressure levels, central obesity and waist-to-hip ratio, depressive symptoms and the mental health component of health-related quality of life. These results reinforce the idea that usefulness of this physical activity programs in primary health care facilities.

Obesity is a major contributor to the development of type 2 diabetes (T2DM), with 80% of individuals being classified as obese. In this line, Andrade-Mayorga et al., illustrate the beauty and complexity the association of perilipin 1 (PLIN1; rs1052700 and rs2304795), lipoprotein lipase (rs283), and adrenoceptor beta 3 (rs4994) polymorphisms with high and low responders (LoRes) to fat mass reduction after 12-weeks of HIIT and dietary energy restriction in 30 adult women with overweight/obese. Their data suggest that rs1052700 (14995A>T) polymorphism of the PLIN1 gene is associated with a differential response to fat mass reduction after a 12-week HIIT intervention. In addition, women with the TT genotype of this genetic variant showed greater changes in fat mass than AA and AT genotypes. However, further studies are needed to confirm these findings. Subsequently, Andrade-Mayorga et al., illustrate the physiological effects and inter-individual variability on fat mass and other health-related and physical performance outcomes after 12 weeks of HIIT in overweight/obese adult women. This intervention caused an improvement in multiple health-related and physical performance outcomes, i.e., reductions in absolute fat mass, body fat percentage, total body mass, blood pressure, and increases in absolute/relative cardiorespiratory fitness. However, beyond the good average group responses found in the present study, a wide range of responses was appreciated in each study variable individually.

Magalhães et al., present the interindividual variability in fat mass loss in response to HIIT with resistance training, moderate continuous training with resistance training (MCT), and control group in adults with T2DM over a 1-year intervention. Their results suggest that the number of fat mass responders did not differ between the MCT or HIIT, compared to the control, following a 1-year exercise intervention in individuals with T2DM. However, low responders to fat mass may still derive reductions in arterial stiffness and structure.

Lastly, Delgado-Floody et al.,  reported improvements in obesity markers, metabolic risk factors, and endurance/muscle performance, and the interindividual variability after 20-weeks of two CT configurations (i.e., HIIT plus resistance training (RT), compared with RT plus HIIT) in 26 women with severe/morbid obesity. Considering the expensive and long treatments before bariatric surgery, the topic of interindividual variability to exercise training is of high interest and value.

Papers in this Research Topic highlight the notion that personalized exercise is a feasible and effective lifestyle modification strategy, for all individuals with, or at risk of, non-communicable chronic diseases. It should be recognized that what is suitable for prevention may be entirely inadequate for treatment, as is also the case with pharmacological management of chronic diseases (Izquierdo et al., 2021). Indeed, in the era of “precision medicine,” it is reasonable to assume that the prescription of exercise as a treatment modality should be individually tailored to the specific characteristics of the patient with respect to program variables (Ramírez-Vélez and Izquierdo, 2019). Concerns have been raised about the true magnitude of response variability as well as maximal trainability. Hypothesized reasons for non-response include insufficient training stimulus (i.e., intensity or specificity of intervention), sex-related differences in response to exercise, and baseline fitness levels. Additionally, the individual interaction of physiological, molecular (i.e., genetics, epigenetics, transcriptomics, and metabolic factors), and environmental factors are being investigated as potential mediators of the lack of a response to exercise in some participants (Izquierdo et al., 2021). Further investigation is warranted to evaluate whether response heterogeneity differs across population subtypes and with similar lifestyle modifications to move closer to a personalized lifestyle medicine that optimizes changes in clinical outcomes based on individual characteristics (Izquierdo et al., 2021). Given the importance of personalized exercise for a healthy aging for today's society, we consider that this eBook will be of great scientific and social impact with the consequent transfer applications. We hope to continue the success of this Research Topic with a third edition in the near future.

## Author Contributions

RR-V and MI: drafted the manuscript. All authors approved the final version.

## Conflict of Interest

The authors declare that the research was conducted in the absence of any commercial or financial relationships that could be construed as a potential conflict of interest.

## Publisher's Note

All claims expressed in this article are solely those of the authors and do not necessarily represent those of their affiliated organizations, or those of the publisher, the editors and the reviewers. Any product that may be evaluated in this article, or claim that may be made by its manufacturer, is not guaranteed or endorsed by the publisher.
